# Evaluation of a novel microfluidic immuno-magnetic agglutination assay method for detection of dengue virus NS1 antigen

**DOI:** 10.1371/journal.pntd.0008082

**Published:** 2020-02-18

**Authors:** Izaskun Alejo-Cancho, Jessica Navero-Castillejos, Aida Peiró-Mestres, Rosa Albarracín, Josep Barrachina, Alexander Navarro, Verónica Gonzalo, Víctor Pastor, José Muñoz, Miguel J. Martínez

**Affiliations:** 1 Department of Clinical Microbiology, Hospital Clinic, Barcelona, Spain; 2 ISGlobal, Barcelona Centre for International Health Research (CRESIB), Hospital Clinic, Universitat de Barcelona, Barcelona, Spain; 3 Department of Tropical Medicine, Hospital Clinic, Barcelona, Spain; DoD - AFHSB, UNITED STATES

## Abstract

**Background:**

Dengue virus (DENV) is the most important arbovirus worldwide, causing infections in endemic countries and returning travellers from these areas. Rapid diagnostic tests are needed to improve patient management and monitor local transmission. The detection of DENV non-structural protein 1 (NS1) is a useful tool for the diagnosis, but the currently available methods can be time consuming or lack sensitivity. The objective of our study was to evaluate a new rapid and semi-quantitative microfluidic DENV NS1 immuno-magnetic agglutination assay based on aggregation of magnetic nanoparticles detected by an electronic reader (Virotrack Dengue Acute and Blubox, Blusense diagnostics, Copenhagen, Denmark).

**Methodology/Principal findings:**

A panel of 135 serum samples from travelers returning from dengue endemic countries was analyzed (74 DENV positive samples including the four DENV serotypes, 26 Zika virus positive samples, 25 chikungunya virus positive samples, 5 malaria positive samples and 5 negative samples). Samples were tested by three different antigen detection methods: SD Dengue NS1 Ag ELISA, SD BIOLINE Dengue Duo and ViroTrack Dengue Acute. The sensitivity observed for SD Dengue NS1 Ag ELISA, ViroTrack Dengue Acute and SD BIOLINE Dengue Duo was 97.2%, 91.1% and 68.1%, respectively. All methods showed high specificity (98.4% for ViroTrack Dengue Acute and 100% for both SD Dengue NS1 Ag ELISA and SD BIOLINE Dengue Duo). SD Dengue NS1 Ag ELISA and ViroTrack Dengue Acute only failed to detect samples positive for DENV-2.

**Conclusions/Significance:**

ViroTrack Dengue Acute is a sensitive and specific assay for DENV NS1 detection. It provides faster results than the ELISA method and a better performance than the rapid immunochromatographic tests. ViroTrack Dengue Acute could represent a valuable tool for rapid diagnosis of DENV infections in returning travellers from endemic countries.

## Author summary

Dengue virus is a human pathogen that causes millions of infections each year. It can be found in nearly all tropical areas, and people living in those regions and travelers who visit them are at risk of disease. The symptoms may vary from a mild flu-like disease to severe hemorrhagic symptoms. Accurate diagnosis of dengue is important for a better management of the disease and for epidemiological surveillance. Detection of DENV antigen is a useful tool to diagnose DENV infections. However, currently available techniques are not rapid (ELISA assays) or not accurate enough (immunochromatographic tests). Here, we evaluate a new antigen detection technique based on aggregation of magnetic nanoparticles. The method combines a short time to results (12 minutes) with a high diagnostic performance. We therefore conclude that this new technique could be a useful tool for the diagnosis of dengue.

## Introduction

Dengue virus (DENV) (genus *Flavivirus*, family *Flaviviridae*) is mainly transmitted by the bites of infected *Aedes* mosquitoes, and there are four different serotypes of the virus (DENV-1, DENV-2, DENV-3 and DENV-4). Infection by one of the DENV serotypes does not induce protection against other serotypes, and secondary infections are associated with a higher risk of severe clinical disease [1] [2]. DENV infection has a large spectrum of clinical manifestations; from asymptomatic infections to a febrile illness (dengue fever) and in a minority of cases a severe life-threatening disease [3]. DENV is considered the most important arthropod borne virus (arbovirus) and the fastest growing vector borne disease worldwide [3]. It is estimated that 390 millions of infections by DENV may occur annually, being 96 millions of them symptomatic [4]. Most of the cases of DENV infection occur in endemic countries. The number of travelers returning from endemic countries is also increasing, and therefore DENV infection has become a common diagnosis among travelers presenting fever at their return[5]. In a surveillance study in returning travelers from 2007 to 2011, DENV infection accounted for 15% of the etiologies of febrile illness[6].

Laboratory diagnosis of DENV infection can be achieved by direct and indirect methods. Direct methods include detection of the viral genome by reverse-transcription polymerase chain reaction (RT-PCR), virus isolation or detection of viral antigens. Indirect diagnosis is based on detection of immunoglobulin (Ig) M and IgG antibodies, and the confirmation by indirect methods is achieved by the observation of seroconversion in paired acute and convalescent serum samples[3]. The detection of non-structural protein 1 (NS1) of DENV, a glycoprotein secreted from infected cells, is a useful tool for the diagnosis of acute DENV infection. NS1 antigen can be detected by enzyme-linked immunosorbent assays (ELISA) or immunochromatographic assays (ICT). Enzyme-linked immunosorbent assays have proven to be a useful tool for NS1 antigen detection, with sensitivity values between 57.7%-95.1% according to different studies[7][8][9][10][11]. However, the ELISA time to results (around 2-3h) and the need for certain laboratory expertise to perform them make them not the best choice for rapid diagnosis. Immunochromatographic tests, on the contrary, are easy to use and provide results in minutes. However, they offer a lower diagnostic performance, with sensitivities ranging from 40% to 79.1% among different reports [9][11]. The specificity observed for both techniques is very high (95%-100%)[7][8][10][11], and therefore a positive NS1 detection represents a laboratory confirmed case of DENV infection (https://ecdc.europa.eu/en/surveillance-and-disease-data/eu-case-definitions).

Immuno-magnetic agglutination (IMA) assays are newly developed methods that use magnetic particles coated with capture molecules (e.g. antibodies, ligands, nucleotides) that bind specifically to the target biomarker, forming clusters that enable the detection. Different assays have been developed for the detection of pathogens, small molecules and proteins[12]. These methods are easy to use and can improve the sensitivity and time to the results of classic methods such as ELISAs [13]. An assay to detect DENV NS1 antigen has been developed based on IMA technology[14].

A rapid and reliable diagnostic test for acute DENV infections would contribute to a better patient management and would be helpful for surveillance programs in order to monitor local virus transmission in non-endemic areas where the vectors are present. The objective of our study was to evaluate a new rapid and semi-quantitative microfluidic DENV NS1 immuno-magnetic agglutination (IMA) assay based on aggregation of magnetic nanoparticles detected by an electronic reader (Virotrack Dengue Acute and Blubox, Blusense diagnostics). The performance of the test was evaluated in samples from acute cases of DENV, chikungunya virus (CHIKV), Zika virus (ZIKV) and *Plasmodium* infections and was compared against established methods for detection of DENV NS1 antigen.

## Materials and methods

### Samples

**Fig 1:**
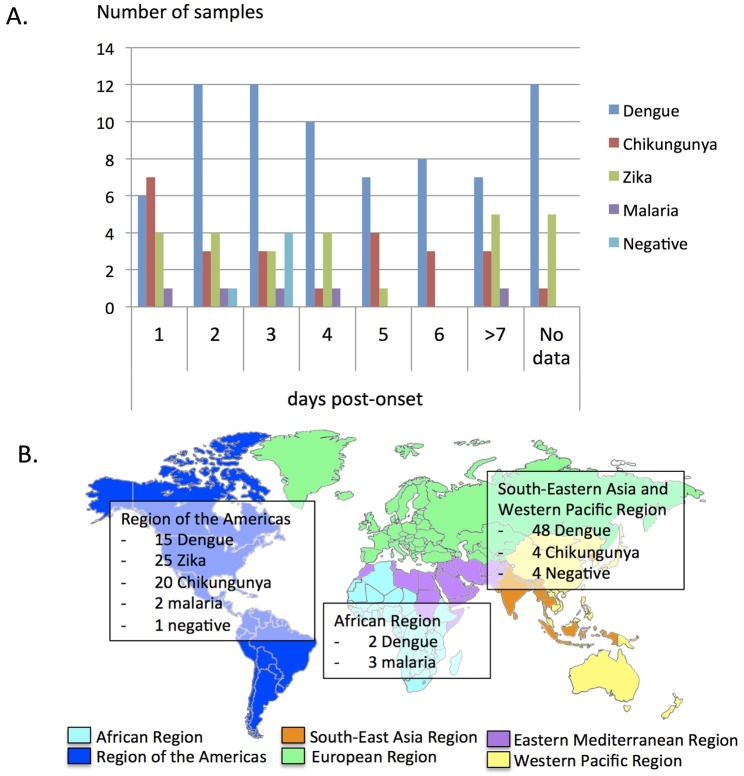
A. Distribution of samples by day post-onset of symptoms and final diagnosis. B. Distribution of samples by visited region, according to the WHO regions. In 11 samples (9 positive DENV samples, one positive ZIKV sample and one positive CHIKV sample) the country visited was not recorded.

A panel of 135 serum samples from travelers returning from dengue endemic countries was analyzed in this study. The panel included 74 DENV positive samples by real time RT-PCR including all DENV serotypes (26 DENV-1, 24 DENV-2, 15 DENV-3, 7 DENV-4, 2 non typed DENV), 26 ZIKV positive samples by real time RT-PCR, 25 CHIKV positive samples by real time RT-PCR, 5 malaria positive samples by thick and thin blood smear and 5 negative samples from patients with fever returning from endemic countries in which an arboviral infection was ruled out. A summary of the samples included in the study, according to days after the onset of symptoms and the geographical area visited by the travelers is shown in Fig 1. The median age of travelers was 35 years old (range 6–74) and 51% were female.

### Laboratory diagnosis

Acute DENV infections in travelers were diagnosed by detection of DENV RNA in serum samples. The following methods were used for detection and/or serotyping of DENV: a commercial real-time RT-PCR (LightMix Modular Dengue, TIB Molbiol, Berlin, Germany, performed on LightCycler 480 II thermal cycler, Roche), an in-house real-time RT-PCR (performed on Stratagene Mx3000P thermal cycler, Thermo Fisher Scienctific)[15] or an in-house generic flavivirus RT-PCR [16]. RT-PCR products obtained with the later method were then visualized in a 2% agarose gel followed by sequencing. The different methods used reflect the availability of diagnostic assays in different periods in our laboratory: the in-house real time RT-PCR was substituted in 2017 by the commercial assay and the generic flavivirus RT-PCR was used for serotyping three samples. Dengue cases were classified as secondary infections when pre-existing immunoglobulin (Ig) G against DENV (measured by PanBio ELISA (Alere, Brisbane, Australia)) were present in the sample positive for DENV RNA [3]. The diagnosis of acute CHIKV and ZIKV infections was achieved by specific real-time RT-PCR (RealStar Chikungunya Virus RT-PCR kit and RealStar Zika Virus RT-PCR kit, Altona Diagnostics GmbH, Hamburg, Germany). Malaria cases were diagnosed by thick and thin blood smears. All samples were stored frozen at -80°C until the DENV NS1 assays were performed.

### Tests for DENV NS1 antigen detection

The samples were tested by three different methods for detection of DENV NS1 antigen: SD Dengue NS1 Ag ELISA (Standard Diagnostic Inc, Kyongii-Do, Korea), SD BIOLINE Dengue Duo (Standard Diagnostic Inc, Kyongii-Do, Korea) and ViroTrack Dengue Acute (BluSense Diagnostics, Copenhagen, Denmark). All assays were performed according to the manufacturer’s instructions.

**Fig 2:**
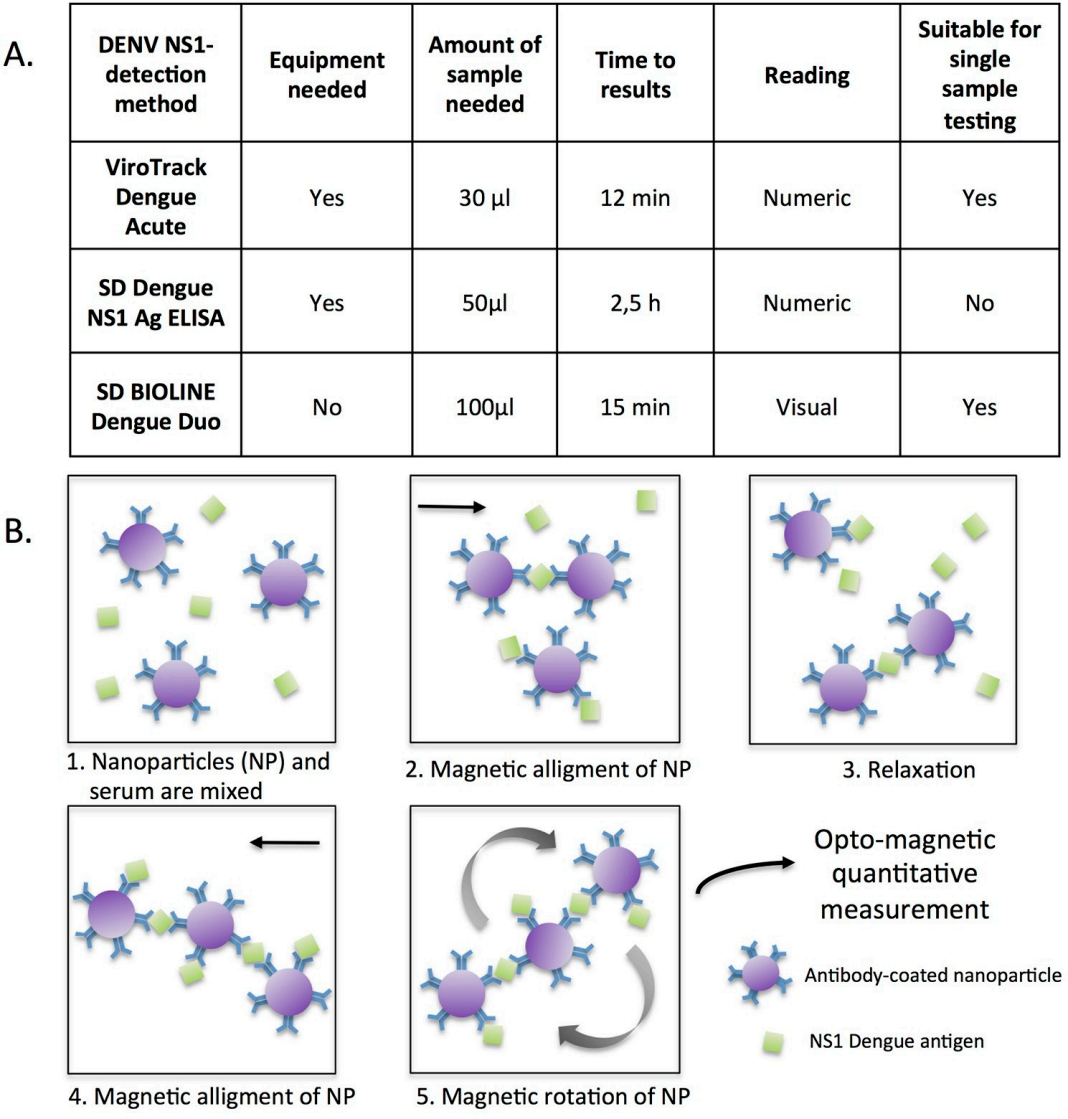
A. Overview of the three DENV NS1 detection methods. B. Schematic principle of the IMA technology. Magnetic nanoparticles coated with monoclonal antibodies against DENV NS1 protein are incubated with the serum sample. Incubation in a strong magnetic field induces NS1-mediated nanoparticle aggregation. The concentration of NS1 in the sample is quantified by measuring the modulation of a transmitted laser light upon magnetic rotation of nanoparticles.

SD Dengue NS1 Ag ELISA (hereinafter also referred to as ELISA) is an enzyme-linked immunosorbent assay for the qualitative detection of NS1 antigen in human serum. SD BIOLINE Dengue Duo kit is a rapid immunochromatographic test (hereinafter also referred to as immunochromatographic test or ICT) that detects both DENV NS1 antigen and antibodies against DENV (IgM/IgG) in human serum, plasma or whole blood. ViroTrack Dengue Acute (hereinafter also referred to as immuno-magnetic agglutination assay or IMA) is a rapid and semi-quantitative microfluidic DENV NS1 detection method based on IMA technology[14]. The IMA test uses magnetic nanoparticles coated with a mix of monoclonal antibodies capable of detecting NS1 from all four DENV serotypes. The test cannot differentiate between the serotypes and returns a positive, negative, or equivocal value relative to the total amount of DENV NS1 antigen (of one or multiple serotypes) present in the sample. The kit consists on a cartridge in which 30 μl of serum, plasma, whole blood or capillary blood are introduced. This cartridge is then inserted on the reader (Blubox; BluSense Diagnostics, Copenhagen, Denmark), in which all the self-contained sample preparation, mixing and reading steps take place. The reading is automatic and quantitative, and the results are obtained on the screen in 12 minutes after loading the cartridge. An overview of the main characteristics of the NS1 detection assays and the principle of the IMA technology is shown in Fig 2.

### Statistical analysis

Sensitivity, specificity, likelihood ratios and agreement values were calculated for all methods having molecular techniques as the gold standard. Sensitivity was calculated as: true positives/ (true positives + false negatives) and specificity as: true negatives/(true negatives + false positives). Confidence interval for sensitivity and specificity were calculated using the efficient-score method described by Newcomb based on the procedure described by Wilson[17]. Likelihood ratios indicate the increase or decrease of probability for a disease for the given test results, and were calculated as follows: positive likelihood ratio (LR+) = sensitivity/(1-specificity); negative likelihood ratio (LR-) = (1-sensitivity)/specificity) and interpreted based on Hayden et al[18]. Kappa coefficient, a measurement of nonrandom agreement between measurements, was also calculated and interpreted following Landis and Koch criteria[19].

### Ethics statement

This study was approved by the Ethical Comitee of Hospital Clinic (File HCB/2018/0931). Leftovers of routine diagnostic serum samples were anonymized and stored frozen until testing.

## Results

All 135 serum samples were tested by ViroTrack Dengue Acute, 133 samples by SD Dengue NS1 Ag ELISA and 130 samples by SD BIOLINE Dengue Duo kit (some samples could not be tested by all methods due to insufficient sample volume).

The sensitivity, specificity, positive and negative likelihood ratios and kappa statistic values obtained are summarized in Table 1. The results obtained for each method by type of pathogen are shown in Table 2. Sensitivity values were higher with the ELISA (97.2%), followed by the IMA assay (91.9%) and the ICT (68.1%). Specificity was 100% for ELISA and ICT and 98.4% for the IMA test. One ZIKV positive sample tested positive in IMA assay. This sample was obtained two days after the onset of the symptoms and tested positive for ZIKV and negative for DENV and CHIKV by real-time RT-PCR. This apparent cross-reactivity was not observed in the other 25 ZIKV positive samples tested.

**Table 1:** Sensitivity, specificity, likelihood ratios and Kappa statistic results for each test with a 95% confidence interval. The gold standard used for the final diagnosis was the DENV RT-PCR result.

	Sensitivity (%, 95% CI)	Specificity (%, 95% CI)	Positive Likelihood ratio	Negative likelihood ratio	Kappa (value, 95% CI)
**ViroTrack Dengue Acute**	91.9 (82.6–96.7)	98.4 (90–99.9)	>10	0.08 (0.04–0.2)	0.9 (0.8–1)
**SD Dengue Ag NS1 ELISA** ^1^	97.2 (89.4–99.5)	100 (92.6–100)	>10	0.03 (0.007–0.11)	1 (0.9–1)
**SD BIOLINE Dengue** **Duo** ^1^	68.1 (55.7–78.5)	100 (90.6–100)	>10	0.36 (0.25–0.52)	0.7 (0.6–0,8)

^1^ Due to insufficient sample volume in DENV positive samples 2 SD Dengue NS1 Ag ELISA and 5 SD BIOLINE Dengue Duo could not be performed.

**Table 2:** Positive results from the total of positive samples tested.

Panel	Number of samples	Positive by ViroTrack Dengue Acute		Positive by SD Dengue NS1 Ag ELISA		Positive by SD BIOLINE Dengue Duo	
		N	%	N	%	N	%
DENV	74	68	91.9	70^1^	97.2	47^2^	68.1
Serotype-1	26	26	100	26	100	20^2^	83,3
Serotype-2	24	19	79.2	21^1^	95,5	14^2^	66.7
Serotype-3	15	15	100	15	100	11	73.3
Serotype-4	7	7	100	7	100	1	1.4
Non-typed	2	1	50	1	50	1	50
ZIKV	26	1	3.8	0	0	0	0
CHIKV	25	0	0	0	0	0	0
Malaria	5	0	0	0	0	0	0
Negative	5	0	0	0	0	0	0

^1^ Due to insufficient sample volume SD Dengue NS1 Ag ELISA could not be performed in 2 DENV-2 samples.

^2^ Due to insufficient sample volume in 2 DENV-1 samples and 3 DENV-2 samples SD BIOLINE Dengue Duo could not be performed.

For all three methods positive likelihood ratios >10 were obtained, which means that a positive result has large effect on post-test probability. For negative likelihood ratio, a ratio <0.1 was obtained both for IMA and ELISA test, which implies a large effect on post-test probability, and a ratio of 0.3 for ICT, which means a small effect on post-test probability.

Both ELISA and IMA methods showed an almost perfect agreement with the real-time RT-PCR gold standard according to the kappa statistic values and Landis and Koch criteria, whereas the ICT showed a substantial agreement.

The ELISA and the IMA assay detected all DENV-1, DENV-3 and DENV-4 positive samples included in the study. Detection rates for DENV-2 positive samples for the ELISA and IMA assays were 95.5% and 79.2%. The tested panel included eight samples from secondary DENV infections: seven were detected by the ELISA, six by the IMA assay and four by the ICT.

Detection rates by days post-onset are shown on Supplementary material (S1 Table), as well as true positive (TP), true negative (TN), false positive (FP) and false negative (FN) values for each test (S2 Table).

## Discussion

DENV infection is a frequent diagnosis in travelers returning from endemic countries[5][6]. The clinical presentation of the infection can range from asymptomatic or mild disease to a severe syndrome. A rapid diagnostic test for dengue could contribute to better patient management and facilitate screening of patients for arboviral surveillance programs in non-endemic areas [20]. In the last years, local transmission of DENV and CHIKV has been documented in several European countries, including the first autochthonous cases in Spain in 2018 [21].

Detection of DENV NS1 protein in serum of infected patients represents the principal antigen-detection diagnostic method for DENV infections. A variety of both ELISAs and ICTs are commercially available. Previous studies have observed sensitivity values of 57.7%-95.1% for ELISA assays[7][8][9][10][11] and 40%-79.1% for immunochromatographic tests[9][11]. While the diagnostic performance of ELISA-based methods is clearly superior to that of immunocromatography-based rapid tests, ELISA methods are more time-consuming requiring 2-3h until the results are obtained. In addition, unlike rapid tests, ELISA-based assays are not as suitable as rapid tests for screening of individual samples, given that they usually require the use of multiple controls in each run. Thus, ELISA-based tests are not optimal for urgent testing of individual samples and the rapid tests, which are suitable for individual sample testing, do not perform sufficiently well.

Immuno-magnetic agglutination assays have been recently developed for the detection of various biomarkers [12][22]. Their simplicity of use and rapid time to results are interesting features for rapid diagnosis of infectious diseases. In this study, we have evaluated a novel IMA assay for detection of DENV NS1 antigen. We compared the IMA assay with an ELISA and with an ICT method, considering the RT-PCR results as the gold standard. On the one hand, it was important to compare the IMA with the ELISA, which represents the reference method for NS1 antigen detection. On the other hand, it was specifically relevant to compare the IMA to the ICT, as both assays represent rapid diagnostic testing and therefore would be used in the same clinical situations.

We show that the sensitivity of the IMA (91.9%) is notably higher than the ICT (68.1%) and only slightly lower than the ELISA (97.2%). The IMA requires the lowest amount of sample, is very simple to perform, provides results in less than 15 minutes and offers a semi-quantitative reading. These features make the IMA a promising candidate for rapid DENV NS1 diagnostic testing.

The IMA technology seems to be not exempt from some limitations that have been described for NS1 detection assays, such as the lower sensitivity for DENV-2 and for secondary dengue infections. DENV-2 was the least detected serotype by all three methods. IMA technology failed to detect the NS1 protein in 6 cases of confirmed DENV infection by RT-PCR, being 5 of them DENV-2 and one non-typed DENV. Limitations in detecting DENV-2 NS1 antigen have already been addressed in other studies[7][8], obtaining a sensitivity of 63% for DENV-2 against a 84% of sensitivity of the other three serotypes pooled[23]. A decrease in sensitivity of NS1 detection kits has also been observed during DENV-2 outbreaks[24].This phenomenon could be related to lower NS1 protein levels in serum in DENV-2 infections[25]. Both ELISA and IMA assays detected all DENV-4 samples, but the ICT only detected one out of seven samples with this serotype. Low sensitivity for the detection of DENV-4 has been previously described for other NS1 assays, including the ICT that was used in our study [11].

Detection of NS1 antigen in secondary DENV infections is challenging because of lower sensitivity, probably due to the formation of immune complexes between NS1 and pre-existing antibodies from the previous DENV infection [25][26]. Other studies have shown a great decrease in sensitivity when comparing primary infections to secondary infections, from 47–71% sensitivity in primary dengue cases to 21–55% in secondary cases with ICT assays[9]. Similar results have been reported for ELISA kits, with sensitivity values dropping from 96.1% to 67.3%[10] in secondary infections. Our study did not include a high number of secondary dengue cases, since the majority of our patients are travelers and DENV infections in travelers are much more likely to be primary infections. Despite the low number of secondary dengue cases analyzed in this study, the IMA technology seems to have better sensitivity than ICT for the diagnosis of secondary infections. A larger study on the diagnostic performance of the IMA technology in endemic areas would be needed to assess the usefulness of the IMA for global dengue diagnostics.

Along with the new test, we also evaluated SD Dengue NS1 Ag ELISA and SD BIOLINE Dengue Duo in our study. The ELISA assay had a very good performance, with results similar to the best results obtained in other studies (sensitivity of 85–95%)[11]. Lower sensitivity values have been observed for ELISA in other studies (60–76%)[9][8]. This difference could be explained by the number of secondary dengue infections, as well as the number of DENV-2 infections included in the studies, two factors that can severely affect the performance of the different kits. Regarding SD BIOLINE Dengue Duo, the sensitivity observed in our study was similar to that observed in other reports (52–66%) [9].

In this study, a sample from a patient with ZIKV infection gave a positive result for ViroTrack Dengue Acute. The rest of the positive ZIKV samples did not give any false positive result by ViroTrack Dengue Acute. This phenomenon of cross reactivity has been previously described in a single sample for other rapid diagnostic tests [27]. Although it is not clear the reasons for this cross reactivity, it seems to be a rare finding.

The IMA test performs automatic reading of the results and quantification, providing a robust assessment of the presence of NS1 antigen in the sample. Although some studies have shown low inter-observer variation in the interpretation of NS1 rapid tests, differences have also been reported and weak positive samples are more prone to be misclassified in ICTs. In conclusion, we evaluated the utility of an IMA technology (ViroTrack Dengue Acute) for the NS1 detection of suspected cases of DENV infection in travelers. The assay compiles several advantages of rapid tests (short time to results, suitability for individual sample testing) and the better performance of ELISA methods. Thus, it could represent a valuable tool for the diagnosis of acute DENV infections.

## Supporting information

S1 Checklist STARD checklist for the reporting of studies of diagnostic accuracy. (DOCX)

S1 Diagram STARD Flow Diagram. (DOCX)

S1 Table Detection rates by days post-onset of symptoms in dengue positive samples. (DOCX)

S2 Table True positives, true negatives, false positives and false negatives for each test. (DOCX)
