# Supplementary material for: Evaluation of a novel microfluidic immuno-magnetic agglutination assay method for detection of dengue virus NS1 antigen
Source: PLoS Negl Trop Dis. 2020 Feb 18;14(2):e0008082. doi: 10.1371/journal.pntd.0008082 (PMC7048294; doi:10.1371/journal.pntd.0008082)
Supplement: S1 Diagram — (DOCX) [file pntd.0008082.s002.docx]

S1 Flow Diagram. STARD Flow Diagram.
