# Supplementary material for: Evaluation of a novel microfluidic immuno-magnetic agglutination assay method for detection of dengue virus NS1 antigen
Source: PLoS Negl Trop Dis. 2020 Feb 18;14(2):e0008082. doi: 10.1371/journal.pntd.0008082 (PMC7048294; doi:10.1371/journal.pntd.0008082)
Supplement: S1 Table — (DOCX) [file pntd.0008082.s003.docx]

**S1 Table. Detection rates by days post-onset of symptoms in dengue positive samples.**

| **Days since onset of symptoms** | **Number of positive samples/total of samples (%)** | | |
| --- | --- | --- | --- |
|  | **ViroTrack Dengue Acute** | **SD Dengue NS1 Ag ELISA** | **SD BIOLINE Dengue Duo** |
| **1** | 5/6 (83,3) | 5/5 (100) | 4/6 (66,7) |
| **2** | 11/12 (91,7) | 12/12 (100) | 8/12 (66,7) |
| **3** | 12/12 (100) | 11/12 (91,7) | 6/11 (54,5) |
| **4** | 8/ 10(80) | 9/9 (100) | 8/10 (80) |
| **5** | 7/7 (100) | 7/7 (100) | 2/6 (33,3) |
| **6** | 7/8 (87,5) | 8/8 (100) | 5/7 (71,4) |
| **>7** | 7/7 (100) | 7/7 (100) | 5/7 (71,4) |
| **NA** | 11/12 (91,7) | 11/12 (91,7) | 9/10 (90) |

NA: not available
