# Supplementary material for: Evaluation of a novel microfluidic immuno-magnetic agglutination assay method for detection of dengue virus NS1 antigen
Source: PLoS Negl Trop Dis. 2020 Feb 18;14(2):e0008082. doi: 10.1371/journal.pntd.0008082 (PMC7048294; doi:10.1371/journal.pntd.0008082)
Supplement: S2 Table — (DOCX) [file pntd.0008082.s004.docx]

**S2 Table. True positives, true negatives, false positives and false negatives for each test.**

|  | **True positives (TP)** | **True negatives (TN)** | **False positives (FP)** | **False negatives (FN)** |
| --- | --- | --- | --- | --- |
| **ViroTrack Dengue Acute** | 68 | 60 | 1 | 6 |
| **SD Dengue NS1 Ag ELISA**^1^ | 70 | 61 | 0 | 2 |
| **SD BIOLINE Dengue Duo**^1^ | 47 | 61 | 0 | 22 |

^1^ Due to insufficient sample volume in DENV positive samples 2 SD Dengue NS1 Ag ELISA and 5 SD BIOLINE Dengue Duo could not be performed.
